# Multicenter validation of PIM3 and PIM2 in Brazilian pediatric intensive care units

**DOI:** 10.3389/fped.2022.1036007

**Published:** 2022-12-14

**Authors:** Daniel Hilário Santos Genu, Fernanda Lima-Setta, José Colleti, Daniela Carla de Souza, Sérgio D’Abreu Gama, Letícia Massaud-Ribeiro, Ivan Pollastrini Pistelli, José Oliva Proença Filho, Thaís de Mello Cesar Bernardi, Taísa Roberta Ramos Nantes de Castilho, Manuela Guimarães Clemente, Cibele Cristina Manzoni Ribeiro Borsetto, Luiz Aurelio de Oliveira, Thallys Ramalho Suzart Alves, Diogo Botelho Pedroso, Fabíola Peixoto Ferreira La Torre, Lunna Perdigão Borges, Guilherme Santos, Juliana Freitas de Mello e Silva, Maria Clara de Magalhães-Barbosa, Antonio José Ledo Alves da Cunha, Marcio Soares, Arnaldo Prata-Barbosa, The Brazilian Research Network in Pediatric Intensive Care (BRnet-PIC)

**Affiliations:** ^1^ Department of Pediatrics, Instituto D’Or de Pesquisa e Ensino, Rio de Janeiro, RJ, Brazil; ^2^ Pediatric Intensive Care Unit, Hospital Assunção, São Bernardo do Campo, SP, Brazil; ^3^ Pediatric Intensive Care Unit, Hospital Sírio Libanês, São Paulo, SP, Brazil; ^4^ Pediatric Intensive Care Unit, Urgências Pediátricas Nova Iguaçu, Nova Iguaçu, RJ, Brazil; ^5^ Instituto de Puericultura e Pediatria Martagão Gesteira, Universidade Federal do Rio de Janeiro, Rio de Janeiro, RJ, Brazil; ^6^ Pediatric Intensive Care Unit, Hospital São Luiz Morumbi, São Paulo, SP, Brazil; ^7^ Pediatric Intensive Care Unit, Hospital e Maternidade Brasil, Santo André, SP, Brazil; ^8^ Pediatric Intensive Care Unit, Hospital São Luiz Jabaquara, São Paulo, SP, Brazil; ^9^ Pediatric Intensive Care Unit, Hospital São Luiz Anália Franco, São Paulo, SP, Brazil; ^10^ Pediatric Intensive Care Unit, Hospital Esperança Olinda, Olinda, PE, Brazil; ^11^ Pediatric Intensive Care Unit, Hospital São Luiz São Caetano, São Caetano do Sul, SP, Brazil; ^12^ Pediatric Intensive Care Unit, Hospital e Maternidade Ribeirão Pires, Ribeirão Pires, SP, Brazil; ^13^ Pediatric Intensive Care Unit, Hospital Santa Helena, Brasília, DF, Brazil; ^14^ Pediatric Intensive Care Unit, Hospital Santa Luzia, Brasília, DF, Brazil; ^15^ Pediatric Intensive Care Unit, Hospital e Maternidade Sino Brasileiro, Osasco, SP, Brazil; ^16^ Department of Research & Development, Epimed Solutions Inc., Rio de Janeiro, RJ, Brazil

**Keywords:** validation study, mortality, intensive care units, pediatric, benchmarking, PIM3, PIM2

## Abstract

**Objective:**

To validate the PIM3 score in Brazilian PICUs and compare its performance with the PIM2.

**Methods:**

Observational, retrospective, multicenter study, including patients younger than 16 years old admitted consecutively from October 2013 to September 2019. We assessed the Standardized Mortality Ratio (SMR), the discrimination capability (using the area under the receiver operating characteristic curve – AUROC), and the calibration. To assess the calibration, we used the calibration belt, which is a curve that represents the correlation of predicted and observed values and their 95% Confidence Interval (CI) through all the risk ranges. We also analyzed the performance of both scores in three periods: 2013–2015, 2015–2017, and 2017–2019.

**Results:**

41,541 patients from 22 PICUs were included. Most patients aged less than 24 months (58.4%) and were admitted for medical conditions (88.6%) (respiratory conditions = 53.8%). Invasive mechanical ventilation was used in 5.8%. The median PICU length of stay was three days (IQR, 2–5), and the observed mortality was 1.8% (763 deaths). The predicted mortality by PIM3 was 1.8% (SMR 1.00; 95% CI 0.94–1.08) and by PIM2 was 2.1% (SMR 0.90; 95% CI 0.83–0.96). Both scores had good discrimination (PIM3 AUROC = 0.88 and PIM2 AUROC = 0.89). In calibration analysis, both scores overestimated mortality in the 0%–3% risk range, PIM3 tended to underestimate mortality in medium-risk patients (9%–46% risk range), and PIM2 also overestimated mortality in high-risk patients (70%–100% mortality risk).

**Conclusions:**

Both scores had a good discrimination ability but poor calibration in different ranges, which deteriorated over time in the population studied.

## Introduction

Mortality predictive models help to assess and compare the performance of pediatric intensive care units (PICUs) over time (1–3). One of the most used, the Pediatric Index of Mortality (PIM), was developed in 1997 and updated in 2003 (PIM2) and 2013 (PIM3) (4–6). External validation studies are needed for use in populations different from the original study, which may differ in the patient profile and, consequently, have different performances (7).

Since its publication, PIM3 has been validated in some countries. However, validation was carried out in studies with a few PICUs or a relatively low number of patients (8–14). In Italy, a study involving 11,109 patients (17 PICUs) demonstrated better performance of PIM3 in predicting mortality and calibration than PIM2 (15). Another study in Belgium, the Netherlands, and Canada (1,428 patients) showed that PIM3 had a lower discriminatory capacity (although good) than the Pediatric Risk of Mortality III score but better calibration (16). In Latin America, only one multicenter study conducted in Argentina (49 PICU; 6,602 patients) concluded that PIM3 underestimated mortality (14). In other low- and middle-income countries, single-center studies in Indonesia (9), India (11, 12), and Colombia (13) found similar results. Recently, one study involving nine hospitals in South Africa (17) and another study in a hospital in Saudi Arabia demonstrated acceptable discrimination but poor calibration (18). In Brazil, there is still a lack of robust evidence on the performance of PIM3. In addition, such models should be reassessed regularly, as they are subject to drift over time (19). This study aimed to validate PIM3 in a large and contemporary sample of patients admitted to Brazilian PICUs and compare its performance with the PIM2 score.

## Materials and methods

### Ethics approval

The study was approved by the Ethics Committee of the coordinator center (D'Or Institute for Research and Education), under the n° 3,384,961 (June 11, 2019), and by the other participating institutions (Supplementary Material - Ethics), which waived informed consent.

### Study design and data setting

This retrospective multicenter cohort study used prospectively collected data from October 2013 to September 2019. We restricted the study to PICUs registered in the Brazilian Research Network in Pediatric Intensive Care (BRnet-PIC) that used the Epimed Monitor® System (Epimed Solutions®, Rio de Janeiro, Brazil), a cloud-based registry for quality improvement, performance evaluation, and benchmarking purposes (20). The number of PICUs increased over time, as they were included in the study from the moment they started recording data on this electronic platform. All patients admitted consecutively younger than 16 years old were included. Readmissions were not excluded and were considered new admissions.

### Data collection

The principal collected data included demographics, admission diagnosis, source of admission, length of stay in the PICU, outcome, and all variables to calculate the PIM3 and PIM2 scores collected within the first hour of PICU admission. Scores were calculated as recommended in the original articles (5, 6). We also recorded the presence of any complex chronic condition (CCC), according to the Feudtner Classification version 2 (21), although this data was not used to calculate the scores.

### Statistical analysis

Continuous variables were described as means or medians, and categorical variables as proportions. We assessed discrimination through the area under the receiver operating characteristic curves (AUROC) and its 95% confidence intervals (CI) and compared the AUROCs using a pairwise evaluation by the Delong method (22). For the calibration assessment across classes of mortality risk, we did not use the most traditionally used method, the Hosmer-Lemeshow goodness of fit (H-L GOF) statistics, due to limitations previously described (23–25). For this reason, we decided to use a new approach to assess calibration: the “calibration belt”. This technique was proposed by the “Italian Group for the Evaluation of Interventions in Intensive Care Medicine (GiViTI)” to investigate the relationship between observed and expected outcomes (26–28). This function results in a real calibration curve that shows the predicted mortality rates on the x-axis and observed mortality rates on the y-axis, plus a CI area around the calibration curve (80% and 95% limits), the “calibration belt”. For this belt, a deviation from the bisector was considered statistically significant when the 95% CI limits did not contain the bisector, the ideal line which indicates a perfect match between the PIM results and the outcomes it tries to predict (23, 25). The mean line of the calibration belt was compared to the bisector using a Wald-like statistic, testing the null hypothesis that there is no difference between this line and the bisector, as previously described (26–28). Standardized mortality ratios (SMR) with 95% CI were calculated by dividing the observed mortality rates by predicted ones. SMR below 1.0 indicates that the model overestimates mortality, while SMR above 1.0 indicates that the model underestimates mortality. Additionally, to assess the performance of the scores over time, we divided the patients into three groups: admitted from October 2013 to September 2015; from October 2015 to September 2017, and from October 2017 to September 2019, and we evaluated the SMR, discrimination, and calibration of PIM3 and PIM2 in these three periods. Statistical analysis was performed using R 4.0.3 (R Foundation for Statistical Computing, Vienna, Austria) and IBM SPSS Statistics, version 24 (IBM Corp., Armonk, NY, United States).

## Results

**Figure 1:**
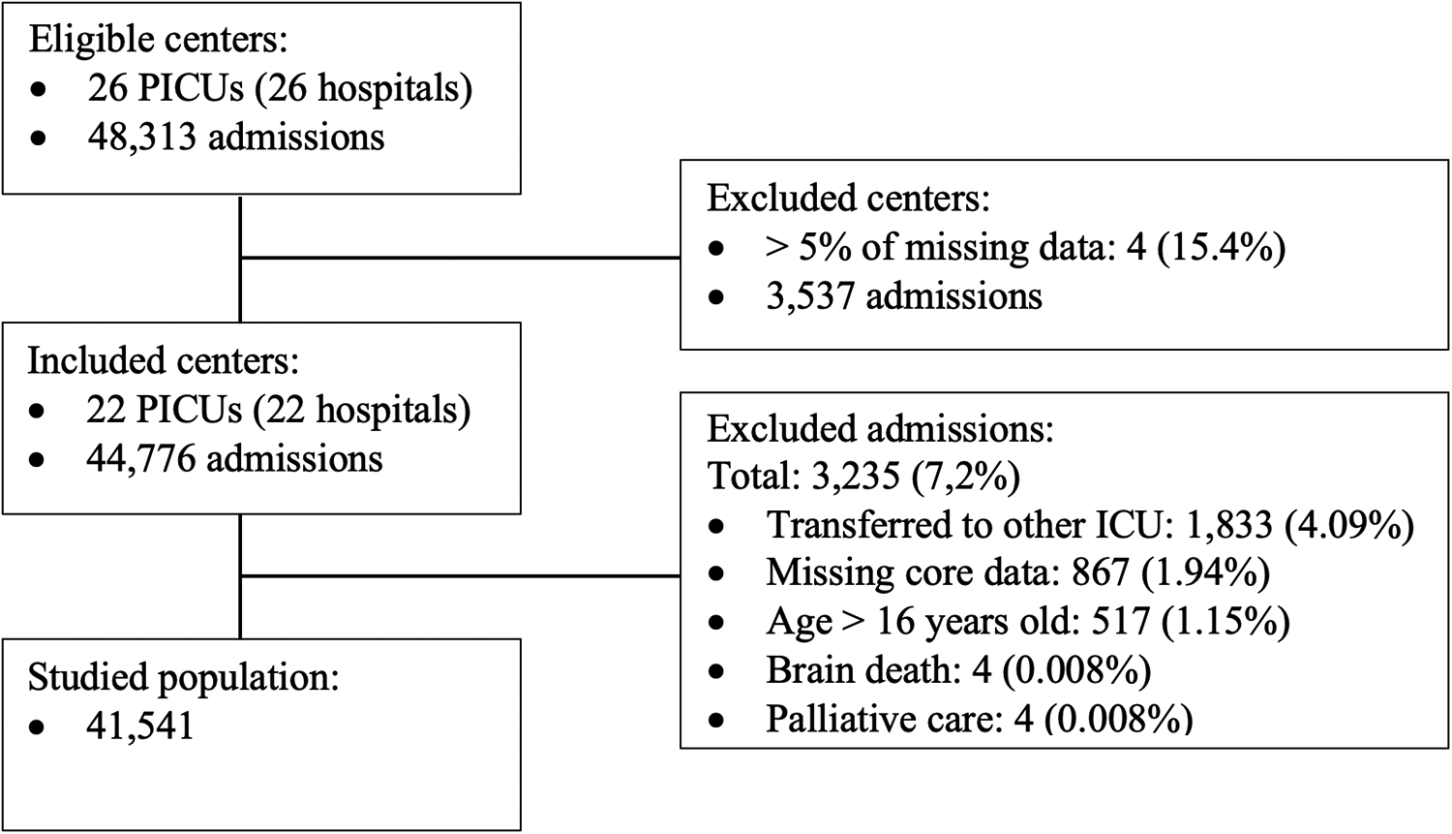
Study flowchart, showing eligibility, exclusion criteria, and final population included in the study.

There were 48,313 eligible patients in 26 PICUs. We excluded four centers (3,537 admissions) because of incomplete medical records that precluded the calculation of any of the PIM scores (> 5% of patients). Twenty-two PICUs remained in the study. Table 1 shows the characteristics of these PICUs. Most of them were private (*n* = 19, 86.4%) and exclusively pediatric (*n* = 14, 63.6%). Of the 44,776 admissions in these 22 PICUs, 3,235 patients (7.2%) were excluded, and 41,541 were included in the study (Figure 1). Table 2 describes the main characteristics of the study population. Most patients aged less than 24 months (58.4%) and were admitted for medical conditions (88.6%). Surgical admissions were mainly for scheduled surgery (59.9%). The origin was the emergency department in 71.1% of cases. Most had respiratory disease (53.8%), followed by neurologic disease (7.7%). CCC was present in 1,607 patients (3.9%); the most common was malignancy (1.0%), followed by gastrointestinal and neurologic/neuromuscular diseases. Upon admission, non-invasive mechanical ventilation was used in 4,378 patients (10.5%), and invasive mechanical ventilation in 2,419 patients (5.8%). The median PICU length of stay was three days (IQR, 2–5).

**Table 1:** Characteristics of the pediatric intensive care units included in the study.

Center No.	City, State	Type of Institution	Type of hospital	Type of PICU	No. of PICU beds	No. of patients in the study	Patients in mechanical ventilation^b^, *n* (%)	Period in the study	Months in the study
1	Rio de Janeiro, RJ	Private	General	Pediatric	24	2162	282 (13.0%)	Oct 2013 – Sep 2019	72
2	Rio de Janeiro, RJ	Private	General	Pediatric	9	1443	127 (8.8%)	Oct 2013 – Sep 2019	72
3	Rio de Janeiro, RJ	Public	Pediatric	Pediatric	11	1456	100 (6.9%)	Oct 2013 – Sep 2019	72
4	Rio de Janeiro, RJ	Private	General	Pediatric	17	1980	124 (6.3%)	Oct 2013 – Sep 2019	72
5	Rio de Janeiro, RJ	Private	Pediatric	Pediatric	13	2145	377 (17.6%)	Feb 2017 – Sep 2019	32
6	Rio de Janeiro, RJ	Private	General	Pediatric	18	2875	730 (25.4%)	Oct 2013 – Sep 2019	72
7	Rio de Janeiro, RJ	Private	General	Pediatric	8	728	210 (28.8%)	May 2017 – Sep 2019	29
8	Rio de Janeiro, RJ	Private	General	Pediatric	16	3576	171 (4.8%)	Oct 2013 – Sep 2019	72
9	São Bernardo do Campo, SP	Private	General	Mixed^a^	11	3185	83 (2.6%)	Oct 2013 – Sep 2019	72
10	Santo André, SP	Private	General	Mixed^a^	27	3055	172 (5.6%)	Oct 2013 – Sep 2019	72
11	São Paulo, SP	Private	General	Pediatric	13	4057	931 (22.9%)	Oct 2013 – Sep 2019	72
12	Ribeirão Pires, SP	Private	General	Mixed^a^	10	1015	109 (10.7%)	Jul 2014 – Sep 2019	63
13	São Paulo, SP	Private	General	Pediatric	11	2866	131 (4.6%)	Oct 2013 – Sep 2019	72
14	São Paulo, SP	Private	General	Mixed^a^	10	3888	1,162 (29.9%)	Oct 2013 – Sep 2019	72
15	São Caetano, SP	Private	General	Pediatric	5	1113	253 (22.7%)	Jun 2017 – Sep 2019	28
16	Osasco, SP	Private	General	Mixed^a^	7	457	103 (22.5%)	Aug 2018 – Sep 2019	14
17	Olinda, PE	Private	General	Mixed^a^	10	2008	246 (12.3%)	Aug 2014 – Sep 2019	62
18	Brasília, DF	Private	General	Mixed^a^	10	666	67 (10.1%)	Oct 2013 – Sep 2019	72
19	Brasília, DF	Private	General	Pediatric	6	759	207 (27.3%)	Aug 2015 – Sep 2019	50
20	Duque de Caxias, RJ	Public	General	Pediatric	20	1306	774 (59.3%)	Oct 2013 – Sep 2019	72
21	Rio de Janeiro, RJ	Public	Pediatric	Pediatric	8	718	414 (57.7%)	Oct 2013 – Sep 2019	72
22	São Paulo, SP	Private	General	Mixed^a^	8	83	24 (28.9%)	Jun 2019 – Sep 2019	4

PICU, pediatric intensive care unit; RJ, rio de Janeiro state; SP, São Paulo state; PE, Pernambuco state; DF, distrito federal (Brazil capital).

^a^Mixed units are pediatric units that also admit newborns.

^b^Non-invasive and/or invasive mechanical ventilation.

**Table 2:** Characteristics of the study population (*n* = 41,541).

Patients’ characteristics	No. (%)
Gender^a^	
Female	18,490 (44.5)
Male	22,765 (54.8)
Age (years), median (IQR)	2 (0–5)
Infant 1 (<12 mo.)	13,811 (33.2)
Infant 2 (12–23 mo.)	10,483 (25.2)
Preschool (2–5 year.)	7,382 (17.8)
Grade schooler (6–12 year.)	8,117 (19.5)
Adolescent (13–16 year.)	1,748 (4.2)
Type of admission	
Medical	36,803 (88.6)
Surgical	4,738 (11.4)
Surgical group, type of admission	
Scheduled surgery	2,840 (59.9)
Emergency surgery	1,898 (40.1)
Source of PICU admission	
Emergency department	29,526 (71.1)
Ward/floor	3,681 (8.9)
Operating room	3,667 (8.8)
Transfer from other hospital	3,002 (7.2)
Other	1,665 (4.0)
Medical group, diagnostic category	
Respiratory	22,335 (53.8)
Neurologic	3,194 (7.7)
Trauma (non-surgical)	1,883 (4.5)
Sepsis	1,331 (3.2)
Cancer	1,111 (2.7)
Gastrointestinal	977 (2.4)
Cardiovascular	607 (1.5)
Other medical admissions	10,103 (24.3)
Presence of CCC (Feudtner)^b^	
All types	1,607 (3.9)
Neurologic and neuromuscular	267 (0.6)
Cardiovascular	147 (0.4)
Respiratory	104 (0.3)
Renal and urologic	113 (0.3)
Gastrointestinal	301 (0.7)
Hematologic or Immunologic	170 (0.4)
Metabolic	12 (0.03)
Other congenital or Genetic defect	58 (0.1)
Malignancy	410 (1.0)
Premature and neonatal	25 (0.06)
Technology dependence	194 (0.5)
Transplantation	0 (0.0)
Support on first hour of admission	
Non-invasive ventilation, *n* (%)	4,378 (10.5)
Invasive mechanical ventilation, *n* (%)	2,419 (5.8)
Vasopressors, *n* (%)	1,130 (2.7)
PICU length of stay (d), median (IQR)	3 (2–5)
Readmissions, *n* (%)	1,917 (4.6)
Elective admissions, *n* (%)	3,814 (9.2)
Deaths, *n* (%)	763 (1.8)

PICU, pediatric intensive care unit; CCC, complex chronic conditions.

^a^Missing, 286 (271 in private, and 15 in public PICUs).

^b^according to Feudtner classification, version 2 (21).

**Figure 2:**
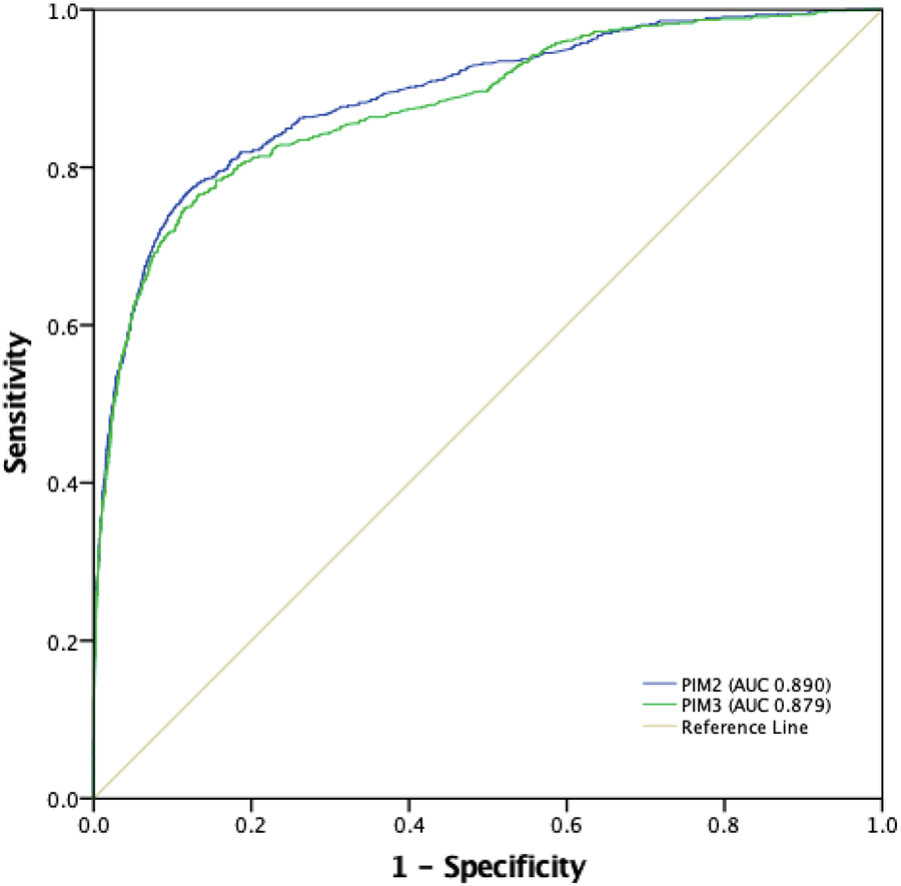
Receiver operating characteristic (ROC) curves for PIM3 and PIM2, and areas under the curve (AUROC). PIM2: 0.89 (95% CI 0.88-0.90); PIM3: 0.88 (95% CI 0.87-0.89) (*P *= 0.0018).

**Figure 3:**
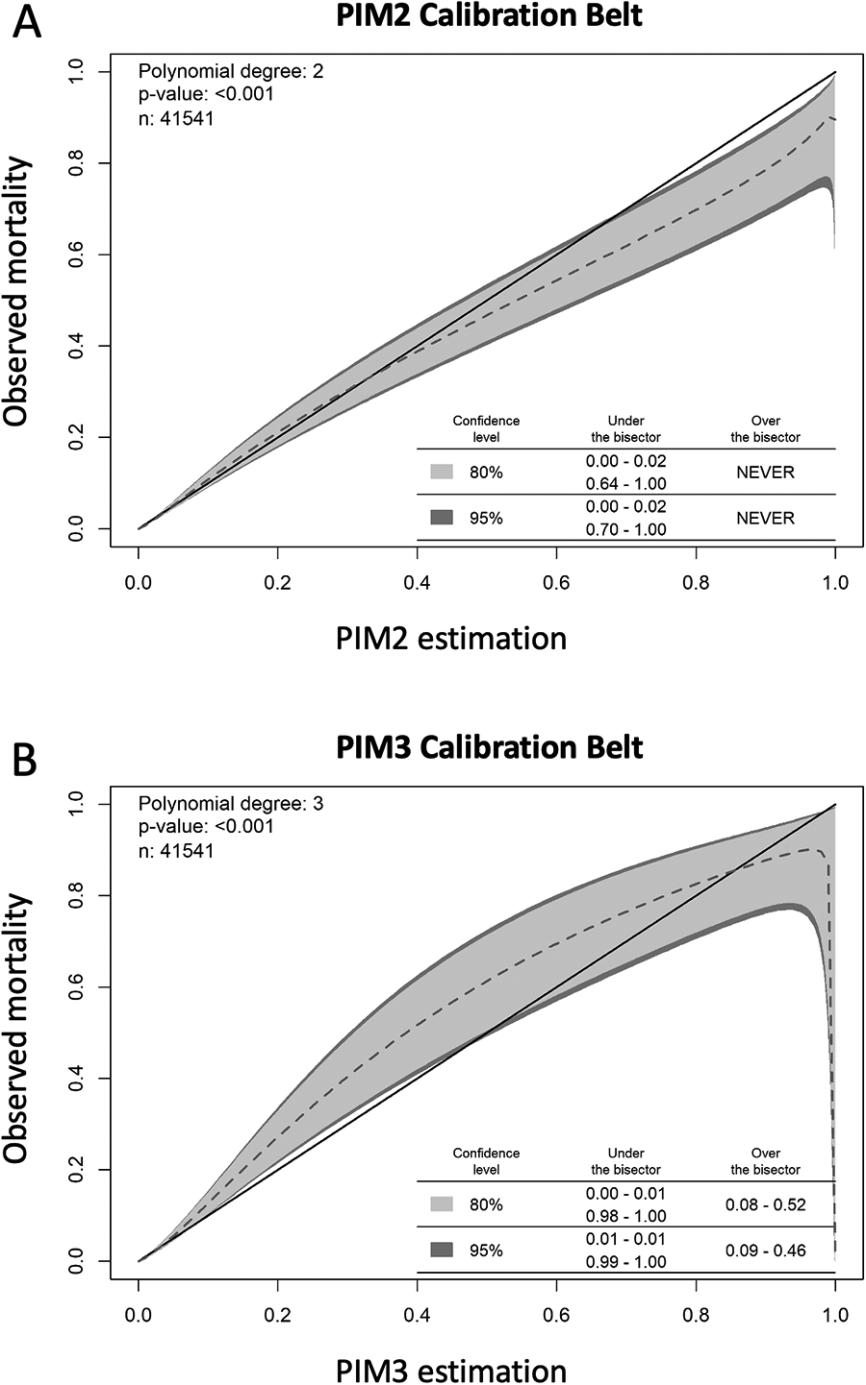
Calibration belts for PIM2 (**A**) and PIM3 (**B**), assessing the concordance of observed vs. expected outcome in 10 deciles of patient risk. The dashed line represents the mean line compared to the bisector, which indicates a perfect match between the PIM results and the outcomes it tries to predict. The *p*-value expresses a Wald-like statistic that tests the null hypothesis that there is no difference between this line and the bisector, which was rejected for PIM2 and PIM3. The inner light gray area marks the 80% CI boundary, and the dark gray outer belt marks the 95% CI boundary. For PIM2, the calibration belt is never over the bisector and is under the bisector (overestimating mortality) in a very low risk range below 2% mortality risk and over 70% predicted mortality, indicating that PIM2 overestimated the mortality for these high-risk patients. For PIM3, the calibration belt is over the bisector between 0.09 and 0.46 predicted mortality, underestimating the mortality in this risk range, and it is under the bisector (overestimating mortality) in a small range in less than 1% and above 99% mortality risk.

Table 3 shows the performance analysis. There were 763 deaths (1.8%). The analysis of subgroups of patients who died showed a greater predominance (compared with the general proportion in the study population) among infants, patients coming from wards/rooms, operating rooms, transferred from other hospitals, and readmissions (Supplementary Material, Table S1). PIM3 predicted 757.2 (SMR 1.008, 95% CI 0.94–1.08), while PIM2 predicted 852.2 (SMR 0.896, 95% CI 0.83–0.96). The discrimination power was good for both scores, 0.88 for PIM3 and 0.89 for PIM2 (Table 3 and Figure 2). The calibration belts are shown in Figure 3. For both scores, the mean line significantly deviated from the bisector (Wald-like statistics, *p*-values < 0.001, Figure 3). When considering the 95% CI, the calibration belt for PIM3 was above the bisector in the predicted mortality range of 9%–46%, demonstrating poor calibration and underestimating mortality in this range. On the other hand, it was below the bisector in two narrow risk ranges, around 1%, and between 99%–100%, overestimating the risk of death only in these ranges. For PIM2, the calibration belt was never above the bisector. Still, it was totally below the bisector in two risk ranges: in the low-risk range of 0 to 2% and the high-risk range of 70 to 100%, overestimating mortality in these ranges. As 90% of the sample had a probability of death <3.3% in both scores (Supplementary Material, Table S2), to facilitate the visualization of the calibration belts in this risk range, we present (Supplementary Material, Figures S1 and S2 ), which only show the risk range from 0 to 5% death probability for both scores. In this range of probability of death, we can see that both scores had a poor calibration between 1% and 3% risk of mortality (1%–2% for PIM2% and 1%–3% for PIM3), overestimating mortality in this group of patients. On the other hand, PIM2 never underestimated mortality in this risk range (it was never above the bisector), but PIM3 started a curve above the bisector around the 5% risk range that went up to 46% (Supplementary Material, Figure S2 and Figure 3). Supplementary Material, Table S2 also shows the study population divided into ten risk groups, with the number of patients, PIM score variation, and observed and expected mortality in each risk group.

**Table 3:** Comparison between the performance of PIM2 and PIM3 in the study population.

Mortality			Discrimination^a^		Calibration^b^	
Observed/Predicted deaths (No.)	SMR (95% CI)	AUROC	95% CI	Under the bisector (95% CI)	Over the bisector (95% CI)
PIM2	763/852.2	0.896 (0.83–0.96)	0.8903	(0.8770–0.9036)	0.00–0.02 0.70–1.00	Never
PIM3	763/757.2	1.008 (0.94–1.08)	0.8793	(0.8652–0.8935)	0.01–0.01 0.99–1.00	0.09–0.46

PIM, pediatric index of mortality; SMR, standardized mortality rate; CI, confidence interval; AUROC, area under the receiver operating characteristic curve.

^a^AUROCs were significantly different by DeLong method: *Z* = 3.1238, *P* = 0.0018, 95% CI AUC difference [0.0041;0.0178].

^b^calibration described as bisector deviation intervals, as proposed by GiViTI (Italian Group for the Evaluation of Intervention in Intensive Care Medicine).

**Figure 4:**
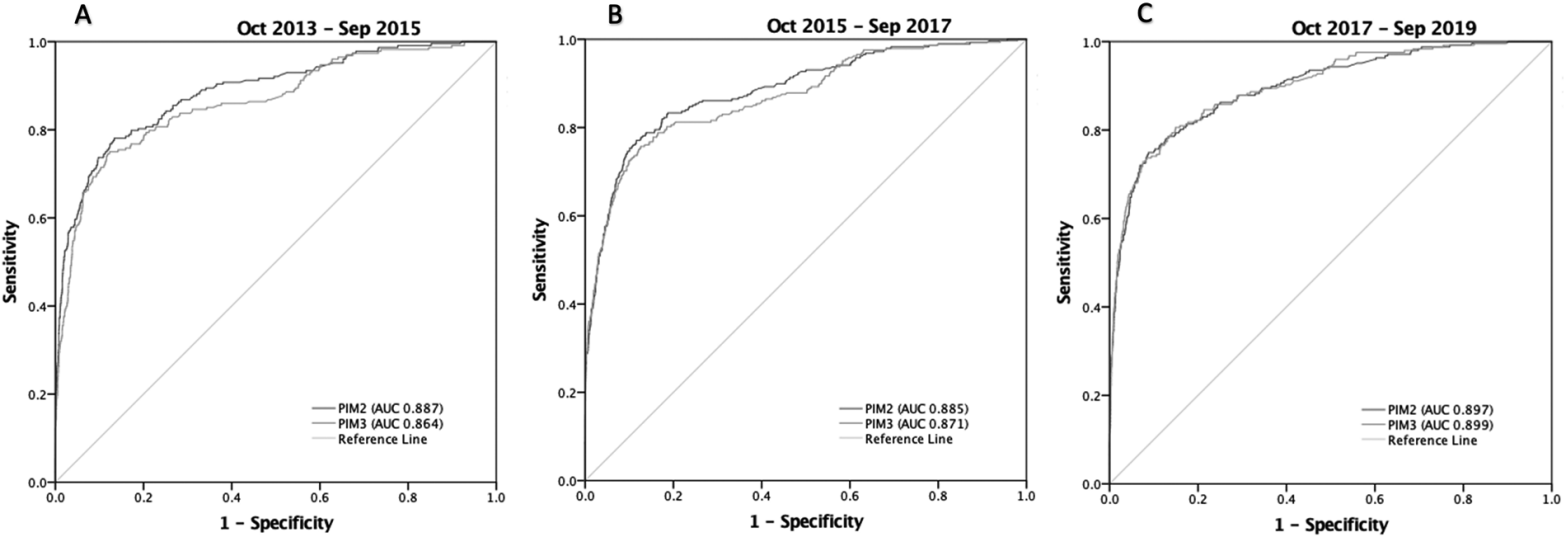
Receiver operating characteristic (ROC) curves for PIM3 and PIM2, and areas under the curve (AUROC) in the three periods from October 2013 to September 2019. (**A**) Period 1(Oct-2013 to Sep-2015): PIM2: 0.89 (95% CI 0.86-0.91); PIM3: 0.86 (95% CI 0.84-0.89) (*P*=0.0018); (**B**) Period 2 (Oct-2015 to Sep-2017): PIM2: 0.89 (95% CI 0.86-0.91); PIM3: 0.87 (95% CI 0.85-0.90) (*P*=0.0136); (**C**) Period 3 (Oct-2017 to Sep-2019): PIM2: 0.90 (95% CI 0.88-0.92); PIM3: 0.90 (95% CI 0.88-0.92) (*P*=0.652).

**Figure 5:**
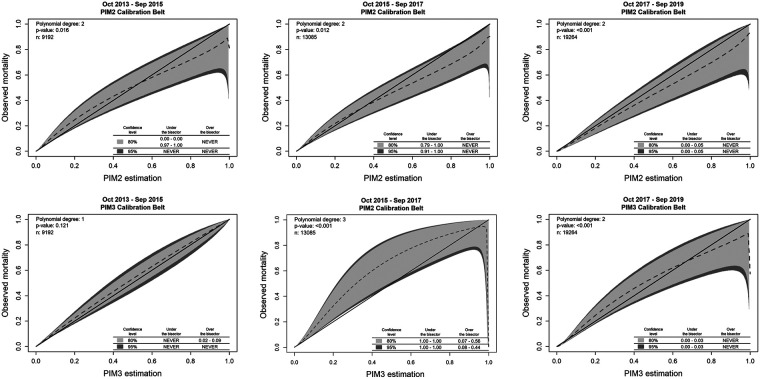
Calibration belts for PIM2 and PIM3 in the three periods from October 2013 to September 2019. PIM2 maintained a good calibration for most risk ranges in all three periods, never underestimating mortality, overestimating it in the second period in the range of more than 91% risk of death and in the third period in the range of 0 to 5% risk of mortality. PIM3, on the other hand, had excellent calibration in the first two years, underestimated mortality in the risk-of-death range of 8%–44% in the second period, and overestimated it in a small range of 0%–3% risk mortality in the last two years.

In assessing the performance of the models over time, PIM3 and PIM2 maintained good discrimination in the three periods, with no difference in the AUROCs in period 3 (Table 4 and Figure 4). As for the standardized mortality ratio, the first four years studied maintained an SMR of around 1 (except for PIM3 in the first biennium, 1.20), showing reasonable adequacy. However, in the last two years, both scores overestimated mortality (PIM2 0.74 and PIM3 0.82) (Table 4). As for calibration, PIM2 maintained a good calibration for most risk ranges in all three periods, never underestimating mortality, overestimating it in the second period in the range of more than 91% risk of death and in the third period in the range of 0 to 5% risk of mortality (Table 4 and Figures 5A–C). PIM3, on the other hand, had excellent calibration in the first two years, underestimated mortality in the risk-of-death range of 8%–44% in the second period, and overestimated it in a small range of 0%–3% risk mortality in the last two years (Table 4 and Figures 5D–F). (Supplementary Material, Table S3) shows the complete statistical data generated by the GiViTI calibration test algorithm.

**Table 4:** Comparison between the performance of PIM2 and PIM3 in the in the study population in the three periods from Oct 2013 to Sep 2019.

Period	Mortality			Discrimination^a^		Calibration^b^	
	Observed/Predicted deaths (No.)	SMR (95% CI)	AUROC	95% CI	Under the bisector (95% CI)	Over the bisector (95% CI)
Oct 2013 to Sep 2015	PIM2	228/236.9	0.99 (0.87–1.13)	0.8868	(0.8617–0.9119)	Never	Never
	PIM3	228/196.6	1.20 (1.05–1.36)	0.8639	(0.8359–0.8920)	Never	Never
Oct 2015 to Sep 2017	PIM2	288/286.3	1.02 (0.91–1.15)	0.8853	(0.8629–0.9077)	0.91–1.00	Never
	PIM3	288/270.0	1.09 (0.96–1.22)	0.8709	(0.8467–0.8950)	1.00–1.00	0.08–0.44
Oct 2017 to Sep 2019	PIM2	247/350.1	0.74 (0.65–0.83)	0.8969	(0.8746–0.9192)	0.00–0.05	Never
	PIM3	247/315.3	0.82 (0.72–0.92)	0.8992	(0.8775–0.9210)	0.00–0.03	Never

PIM, pediatric index of mortality; SMR, standardized mortality rate; CI, confidence interval; AUROC, area under the receiver operating characteristic curve.

^a^AUROCs were significantly different by DeLong method in period 1 (*Z* = 3.1146, *P* = 0.0018, 95% CI AUC difference [0.0085;0.0373]) and in period 2 (*Z* = 2.4686, *P* = 0.0136, 95% CI AUC difference [0.0030;0.0259]), but not in period 3 (*Z* = −0.45098, *P* = 0.652, 95% CI AUC difference [-0.012;0.008]).

^b^calibration described as bisector deviation intervals, as proposed by GiViTI (Italian Group for the Evaluation of Intervention in Intensive Care Medicine).

## Discussion

This study evaluated the performance of PIM2 and PIM3 in 41,541 patients admitted from 2013 to 2019 in 22 Brazilian PICUs. Both scores had a good capacity to discriminate between survivors and non-survivors, but the PIM3 showed overall better performance when evaluated only by the standardized mortality rate. However, in calibration, both overestimated mortality in the low-risk range, where more than 90% of the population studied was concentrated. PIM3 tended to underestimate mortality in medium-risk patients, and PIM2 tended to overestimate mortality in low and high-risk patients. Both scores had excellent calibration in the first two years studied, decreasing in the following four years. To our knowledge, this is the most extensive external validation study of the PIM3.

In Brazil and Latin America, PIM2 (29–35) and PIM3 (13, 14, 36, 37) validation studies were conducted in a single or a few centers and included few patients, except for the 2018 study in Argentina (14). They generally showed good discrimination but poor calibration. In these studies, PIM2 tended to underestimate mortality (29, 31, 33, 34) but overestimated in one (30). PIM3 also had good discrimination but poor calibration (13, 14, 36, 37). Only one study reported that PIM2 had good calibration using the H-L GOF test (35).

Our study population had very different characteristics from the studies that initially developed and validated PIM2 and PIM3. These had a large percentage of cardiac patients (25.5% and 26.1%), non-cardiac postoperative patients (19.2% and 21.1%), and a high percentage of trauma in the PIM2 study (9.3%). Patients with respiratory problems accounted for only 21.6% and 25.1%, respectively. In contrast, respiratory diseases accounted for 53.8% of our sample and heart disease only 1.5%. Another difference with the original PIM3 validation study was that our frequency of elective admissions was lower (9.2% vs. 41.0%), as were admissions for recovery from procedures (6.8% vs. 39.7%). In our cohort, mortality was 1.8%, a rate lower than that described in the original study (4.1% in the United Kingdom/Ireland and 2.8% in Australasia) (6). However, in our sample, among patients on invasive mechanical ventilation in the first hour, the mortality was 7.8% (9.1% for any type of ventilation), values closer to the original study of PIM3, whose mortality in this subgroup was 5.9% (United Kingdom/Ireland) and 4.8% (Australasia). Such differences can be due to the type of units (general vs. cardiac/surgical) and differences in the patient profile. The higher proportion of deaths of patients from rooms/wards, other hospitals, surgical centers, and readmissions suggests that these subgroups arrived at the ICU in worse conditions and may represent typical characteristics of these hospitals in Brazil. In our understanding, this reinforces the importance of external validation studies like ours. Still, those differences do not necessarily indicate the need to recalibrate the scores to the local setting (38).

In our sample, the SMR for PIM2 was below 1.0, indicating fewer deaths than predicted. For PIM3, it was 1.0. But looking at the SMR over time, we notice that PIM2 was more stable in the first four years (SMR around 1.0), overestimating mortality in the last two years. On the other hand, PIM3 had a progressive decline in SMR. A similar result was found by Quiñónez et al. (13). In their study, the SMR was 1.00 for PIM3 and 0.66 for PIM2. Also, the AUROC was 0.89 and 0.87, respectively, but the H-L GOF test suggested that only PIM3 had adequate calibration. In contrast, PIM3 underpredicted mortality in the other studies already cited (9, 10, 14). In any case, in our study, there was a marked reduction in the SMR for the PIM3 in the last six years (from 1.20 to 0.82). Although this is relatively expected, the intensity of the fall may be associated with some possibilities. Many participating hospitals are new. The natural maturation of teams and care processes may have positively impacted results. On the other hand, almost all hospitals participate in international accreditation programs. The PICUs managers are challenged in monthly meetings to present proposals to improve their indicators, one of them is SMR. This performance improvement may reflect a continuous improvement of processes, sentinel events handling, and hospital infrastructure investment.

Regarding the discrimination capacity, both scores had a good overall performance in our study, although we must consider that the sample had very unbalanced classes: almost 50:1 ratio of survivors vs. non-survivors. PIM2 had better discrimination power in the first four years, and PIM3 had better performance in the third study period. Two studies had similar results (12, 36).

As for calibration, the mean calibration line (without confidence intervals) for PIM2 and PIM3 deviated significantly from the ideal curve (bisector). However, the calibration belt proposed by the GiViTI group (26, 27) considers the 95% confidence interval of the calibration curve to assess the calibration. In this original approach in pediatric studies, only used in one study (16), both scores showed calibration problems, mainly in the low-risk range (0%–3%), overestimating mortality in this sample. PIM2 also overestimated mortality in the high-risk ranges (70 to 100%) but never underpredicted mortality. On the other hand, PIM3 underestimated mortality in the medium-risk range (9%–46%). These findings may have resulted from differences in the profile of the population studied in relation to the original study. Still, they may also be a result of the consolidation of results from the long period of the study.

In fact, we were able to demonstrate that the calibration varied over time. In the first two-year study period, both scores had excellent calibration. In the second period, PIM2 overestimated mortality in the high-risk range, and PIM3 underestimated in the medium-risk range. In the last period, both scores overestimated mortality in the low-risk range. This miscalibration may reflect an inadequate fit of the sample case mix or be explained by the tendency of the score calibration to drift over time (24).

Calibration was investigated in all but one study (16) using the H-L GOF test. Unlike classic statistics, in the H-L, a *p*-value ≥ 0.05 (i.e., not significant) indicates good calibration (24). It becomes a problem in a model with a large sample size (> 25,000), like ours, because the model can be misinterpreted as poorly calibrated even when it is not (23, 24, 28), which would happen in our study if we had used the H-L. Another criticism is the fact that the H-L result does not indicate the risk classes affected by the deviations between the observed and predicted mortality, as well as the direction of these deviations (23, 26). This has been mitigated by the use of calibrations plots between predicted and observed mortality, but these graphs are not precisely a curve, representing independent associations in each risk group; that is, the connection between the points is made only to simulate a curve, but there are no actual values in those intervals (26, 27). When using the calibration belt, the confidence intervals are calculated and plotted, allowing secure information about the statistical significance of the calibration across the entire severity spectrum (0%–100%). This can be a great advantage when populations are very different from those in which the score was developed, like ours.

One limitation of our study is that the sample was composed mainly of private hospitals and a predominance of PICUs from the southeastern region of Brazil, the most developed, which may not be representative of the entire country. However, this region concentrates most of the population and PICUs. According to the last census of the Brazilian Association of Intensive Care Medicine (39), there are 613 PICUs in Brazil, with 4,380 beds. Most are in the southeastern region, which has 52.4% of the beds. The private sector has 50% of the available beds. The country is unequal, and in large cities such as Rio de Janeiro and São Paulo, access to the private network reaches almost 50%. Still, this percentage is between 8%–22% in most of the country. Other studies evaluating a large number of patients exclusively from public PICUs in Brazil would be welcome.

In conclusion, this study showed that both scores had a good performance at discrimination ability but a poor calibration, which deteriorated over time in the population studied.

## Data Availability

The original contributions presented in the study are included in the article/**Supplementary Material**. Further inquiries can be directed to the corresponding author.
