# Supplementary material for: Multicenter validation of PIM3 and PIM2 in Brazilian pediatric intensive care units
Source: Front Pediatr. 2022 Dec 14;10:1036007. doi: 10.3389/fped.2022.1036007 (PMC9795232; doi:10.3389/fped.2022.1036007)
Supplement: Supplementary file 1 [file Datasheet1.pdf]

Supplementary Material

Performance of the PIM3 and PIM2 in 41,541 patients admitted to 22 Brazilian Pediatric Intensive Care Units

Daniel Hilário Santos Genu, MD, MSc[1]; Fernanda Lima-Setta, MD, PhD[1]; José Colleti Jr, MD, PhD[2]; Daniela Carla de Souza, MD, PhD[3]; Sérgio D'Abreu Gama, MD[4]; Letícia Massaud Ribeiro, MD, MSc [5]; Ivan Pollastrini Pistelli, MD, PhD[6], José Oliva Proença Filho, MD[7]; Thaís de Mello Cesar Bernardi, MD[8]; Taísa Roberta Ramos Nantes de Castilho, MD[9]; Manuela Guimarães Clemente, MD[10]; Cibele Cristina Manzoni Ribeiro Borsetto, MD[11]; Luiz Aurelio de Oliveira, MD[12]; Thallys Ramalho Suzart Alves, MD[13]; Diogo Botelho Pedroso, MD[14]; Fabíola Peixoto Ferreira La Torre, MD[15]; Lunna Perdigão Borges[16]; Guilherme Santos[16]; Juliana Freitas de Mello e Silva, PhD[1]; Maria Clara de Magalhães-Barbosa, MD, PhD[1]; Antonio José Ledo Alves da Cunha, MD, PhD[1,5]; Marcio Soares, MD, PhD[1,16]; Arnaldo Prata-Barbosa, MD, PhD[1,5], on behalf of the Brazilian Research Network in Pediatric Intensive Care (BRnet-PIC).

1 Supplementary Material - Ethics

The study protocol was approved by the following Institutional Review Boards:

1) Research Ethics Committee of the "D'Or Institute for Research and Education (IDOR)", under the **nº 3.384.961**, which was responsible for the following hospitals, all from the D'Or Network of hospitals in Brazil:
    a. in Rio de Janeiro, Brazil: Hospital Caxias D'Or, Hospital Copa D'Or, Hospital Oeste D'Or, Hospital Quinta D'Or, Hospital Real D'Or, Hospital Rios D'Or, Hospital Jutta Batista, Hospital Estadual da Criança, and Hospital Adão Pereira Nunes;
    b. in São Paulo, SP: Hospital Ribeirão Pires, Hospital São Luiz São Caetano, Hospital São Luiz Anália Franco, and Hospital Sinobrasileiro;
    c. in Brasília, DF: Hospital Santa Luzia and Hospital Santa Helena;
    d. in Olinda, PE: Hospital Esperança.
2) Research Ethics Committee of the "Hospital e Maternidade São Luiz)", under the **nº 3,558,506**, which was responsible for the following hospitals in São Paulo, Brazil: Hospital Brasil, Hospital São Luiz Jabaquara, and Hospital São Luiz Morumbi.
3) Research Ethics Committee of the Hospital Assunção, São Bernardo do Campo, SP, Brazil, under the **nº 3,805,463**.
4) Research Ethics Committee of the Hospital Sirio Libanês, São Paulo, SP, Brazil, under the **nº 3,573,580**.
5) Research Ethics Committee of the Instituto de Puericultura e Pediatria Martagão Gesteira, of the Federal University of Rio de Janeiro, Brazil, under the **nº 3,707,277**.

2 Supplementary Tables

TABLE S1 | Characteristics of the total study and patients who died.

	Total sample	Deaths			
**Patients' characteristics**	**No. (%)**	**Absolute No.**	**%, total sample**	**%, by category**	**%, among deaths**
No. of patients	41,541 (100.0)	763	1,84		
Gender					
Female	18,490 (44.5)	346	0.83	1.87	45.35
Male	22,765 (54.8)	413	0.99	1.81	54.13
Missing	286 (0.7)	4	0.01	1.40	0.52
Age (yrs), median (IQR)	2 (0-5)	1 (0-5)			
Infant 1 (<12 mo.)	13,811 (33.2)	325	0.78	2.35	42.60
Infant 2 (12-23 mo.)	10,483 (25.2)	176	0.42	1.68	23.07
Preschool (2-5 yr.)	7,382 (17.8)	79	0.19	1.07	10.35
Grade schooler (6-12 yr.)	8,117 (19.5)	143	0.34	1.76	18.74
Adolescent (13-16 yr.)	1,748 (4.2)	40	0.10	2.29	5.24
Type of admission					
Medical	36,803 (88.6)	657	1.58	1.79	86.11
Surgical	4,738 (11.4)	106	0.26	2.24	13.89
Surgical group, type of admission					
Scheduled surgery	2,840 (59.9)	64	0.15	2.25	8.39
Emergency surgery	1,898 (40.1)	42	0.10	2.21	5.50
Source of PICU admission					
Emergency department	29,526 (71.1)	337	0.81	1.14	44.17
Ward/floor	3,681 (8.9)	134	0.32	3.64	17.56
Operating room	3,667 (8.8)	84	0.20	2.29	11.01
Transfer from other hospital	3,002 (7.2)	148	0.36	4.93	19.40
Other	1,665 (4.0)	60	0.14	3.60	7,86
Support on first hour of admission					
Non-invasive ventilation, n (%)	4,378 (10.5)	91	0.22	2.08	11.93
Invasive mechanical ventilation, n (%)	2,419 (5.8)	528	1.27	21.83	69.20
Non-invasive/Invasive ventilation, n (%)	6,797 (16.3)	619	1.49	9.11	81.13
Vasopressors, n (%)	1,130 (2.7)	335	0.81	25.57	43.91
PICU length of stay (d), median (IQR)	3 (2-5)	7 (2-22)			
Readmissions, n (%)	1,917 (4.6)	99	0.24	5.16	12.98
Elective admissions, n (%)	3,814 (9.2)	88	0.21	2.31	11.53

TABLE S2 | Observed and expected number of deaths and survivors across ten groups of risk.

	PIM 3						PIM 2					
			Deaths		Survivors				Deaths		Survivors	
	No. of patients	PIM3 (%)	Obs.	Exp.	Obs.	Exp.	No. of patients	PIM2 (%)	Obs.	Exp.	Obs.	Exp.
1	3708	0.00-0.15	5	5,1	3703	3702,89	4206	0.00-0.18	6	6.9	4.330	4329.1
2	4601	0.15-0.17	4	7,2	4597	4593,81	4123	0.18-0.24	11	9.2	4.259	4260.8
3	4154	0.17-0.23	7	8,2	4147	4145,85	4166	0.24-0.36	15	13.5	4.211	4212.5
4	4156	0.23-0.39	16	12,8	4140	4143,17	4658	0.36-0.75	30	27.3	4.351	4353.7
5	4202	0.39-1.21	47	28,2	4155	4173,78	3989	0.75-0.86	21	33.9	4.103	4172,4
6	4143	1.21-1.26	19	50,9	4124	4092,09	3925	0.86-0.99	35	38.3	4.024	4026,5
7	4188	1.26-1.42	23	55,9	4165	4132,11	4012	0.99-1.19	41	47.9	4.292	4197,0
8	4081	1.42-1.78	28	64,3	4053	4016,73	4154	1.19-1.59	65	57.4	4.031	4170,4
9	4159	1.78-3.28	74	97,8	4085	4061,16	4163	1.59-3.33	95	97.4	4.132	4118,5
10	4149	3.28-100.0	540	426,8	3609	3722,19	4145	3.33-100.0	467	541.6	3.758	3562,8

TABLE S3 | Statistical data from the GiViTI calibration test.

Calibration belt	statistic	p-value	β_0	β_1	β_2	β_3
PIM2 (total period, 0-100%)	55.742	7.078e-11	-0.12986053	0.77822534	-0.05761306	0
PIM3 (total period, 0-100%)	74.314	< 2.2e-16	0.461063853	0.934334733	-0.091653228	-0.009438559
PIM2 (total period, 0-5%)	48.975	9.48e-0.8	19.9075507	13.8708989	2.6723262	0.1818811
PIM3 (total period, 0-5%)	80.543	< 2.2e-16	41.6931481	28.8661606	5.9649037	0.4114314
PIM2 Period 1	15.413	0.01584	0.03746302	0.73960160	-0.06350381	0
PIM3 Period 1	4.2317	0.1205	0.1320599	0.9917300	0	0
PIM2 Period 2	16.012	0.01212	-0.14361229	0.74131244	-0.05240984	0
PIM3 Period 2	50.442	4.655e-08	0.84031820	1.03797804	-0.08700228	-0.01185569
PIM2 Period 3	56.811	4.199e-11	-0.21517607	0.84446101	-0.05802483	0
PIM3 Period 3	55.877	6.625e-11	0.17337021	0.81340745	-0.08691097	0

Period 1: 2013-10-01 to 2015-09-30; Period 2: 2015-10-01 to 2017-09-30; Period 3: 2017-10-01 to 2019-09-30

3 Supplementary Figures

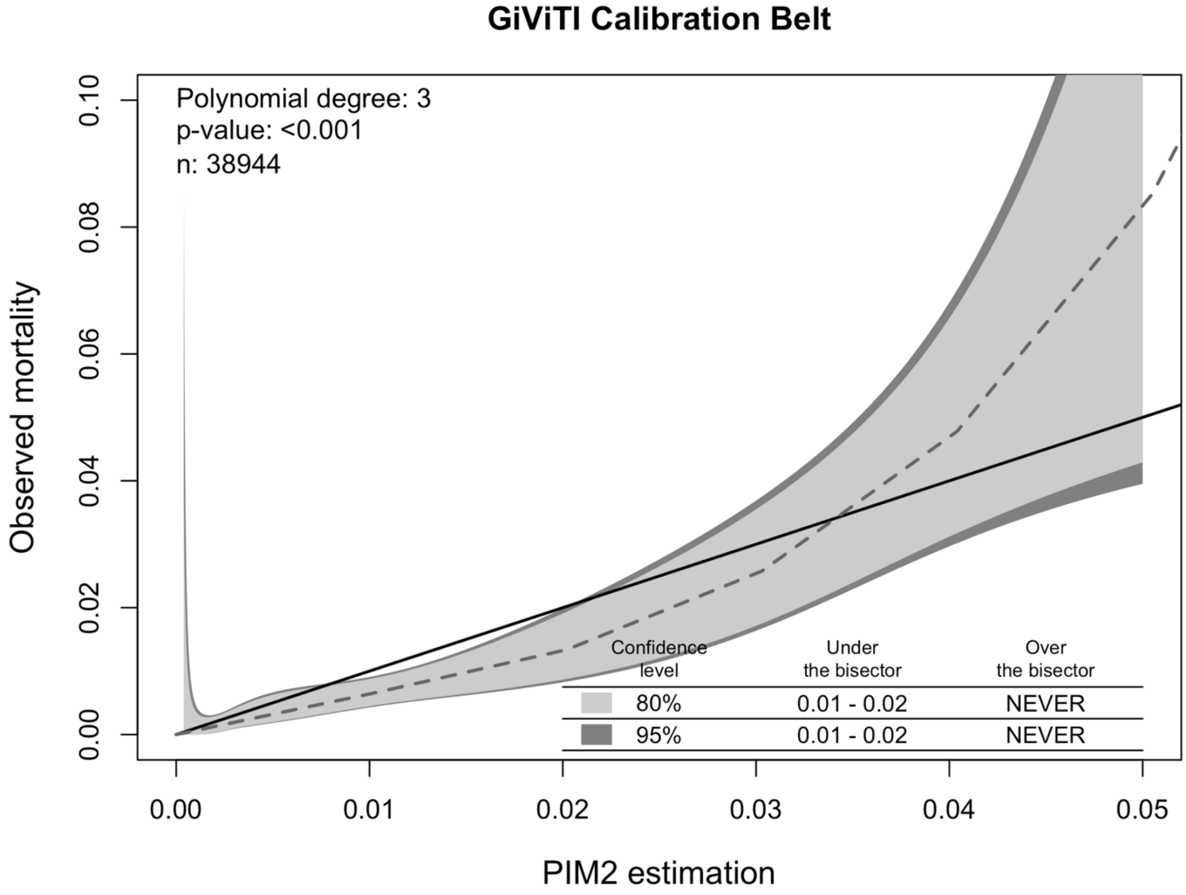

Figure S1. The PIM2 calibration belt for the risk range between 0 and 5% (about 94% of the population). The dashed curve represents the mean line compared to the bisector, which indicates a perfect match between the PIM2 results and the outcomes it tries to predict. The p-value expresses a Wald-like statistic that tests the null hypothesis that there is no difference between this line and the bisector, which was rejected. The belt (95% confidence interval) had adequate calibration in most of this risk range, never underestimating mortality, but a poor calibration between a small range between 1 and 2% risk of mortality, overestimating mortality in this group of patients.

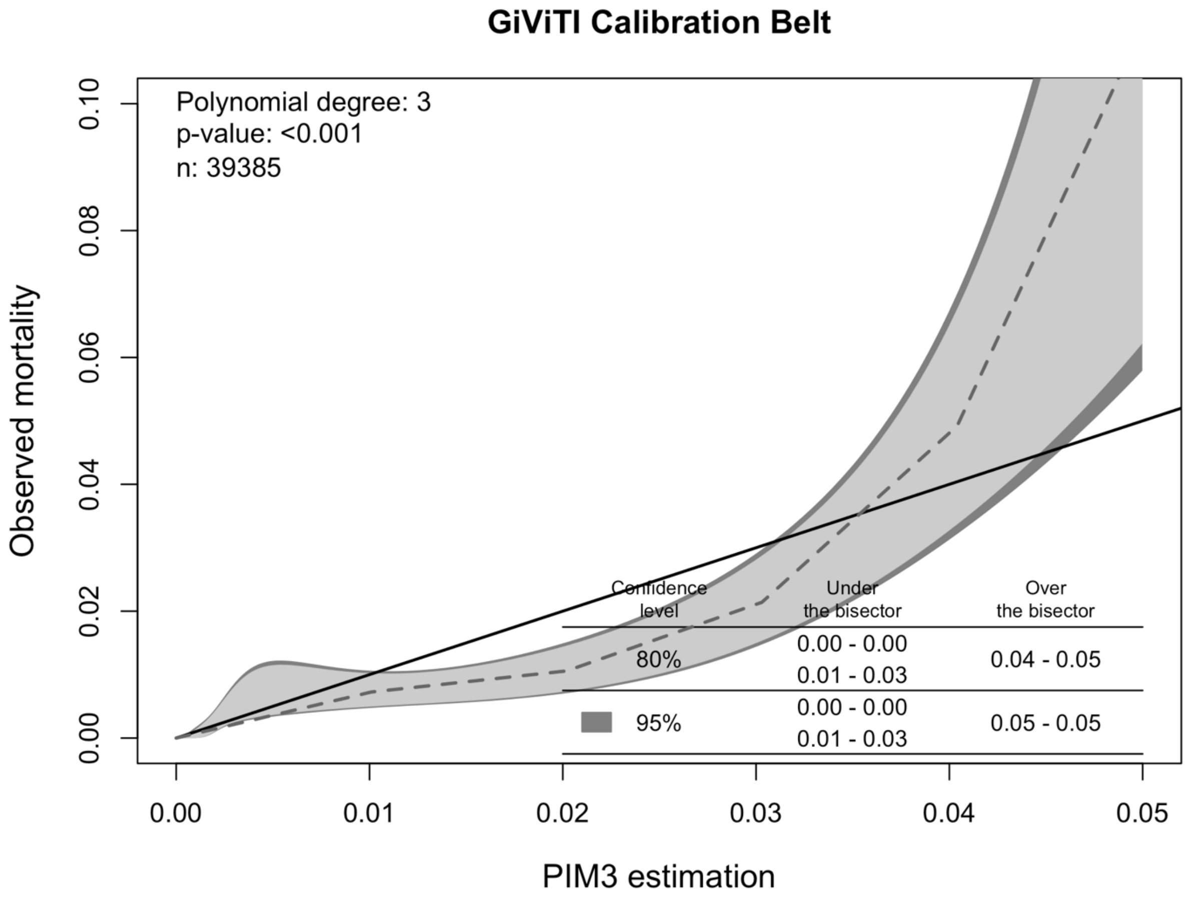

Figure S2. The PIM3 calibration belt for the risk range between 0 and 5% (about 95% of the population). The dashed curve represents the mean line compared to the bisector, which indicates a perfect match between the PIM3 results and the outcomes it tries to predict. The p-value expresses a Wald-like statistic that tests the null hypothesis that there is no difference between this line and the bisector, which was rejected. The belt (95% confidence interval) had a poor calibration between 1 and 3% risk of mortality (overestimating mortality in this group of patients) and above 4.5% (underestimating mortality). Calibration was only adequate in the range of 0-1% and 3-4.5% mortality risk.
